# In vitro triple coculture with gut microbiota from spondyloarthritis patients is characterized by inter-individual differences in inflammatory responses

**DOI:** 10.1038/s41598-022-13582-7

**Published:** 2022-06-21

**Authors:** Annelore Beterams, Marta Calatayud Arroyo, Kim De Paepe, Ann-Sophie De Craemer, Dirk Elewaut, Koen Venken, Tom Van de Wiele

**Affiliations:** 1 Center for Microbial Ecology and Technology (CMET), Department of Biotechnology, Ghent University, Coupure Links 653 Building A, 9000 Ghent, Belgium; 2 Department of Internal Medicine and Pediatrics, Division of Rheumatology, Ghent University Hospital, Ghent, Belgium; 3 Unit for Molecular Immunology and Inflammation Unit, VIB-UGent Center for Inflammation Research, Ghent, Belgium

**Keywords:** Ankylosing spondylitis, Bacterial host response

## Abstract

Spondyloarthritis is a group of chronic inflammatory diseases that primarily affects axial or peripheral joints and is frequently associated with inflammation at non-articular sites. The disease is multifactorial, involving genetics, immunity and environmental factors, including the gut microbiota. In vivo*,* microbiome contributions are difficult to assess due to the multifactorial disease complexity. In a proof-of-concept approach, we therefore used a triple coculture model of immune-like, goblet and epithelial cells to investigate whether we could detect a differential impact from spondyloarthritis- vs. healthy-derived gut microbiota on host cell response. Despite their phylogenetic resemblance, flow cytometry-based phenotypic clustering revealed human-derived gut microbiota from healthy origin to cluster together and apart from spondyloarthritis donors. At host level, mucus production was higher upon exposure to healthy microbiota. Pro-inflammatory cytokine responses displayed more inter-individual variability in spondyloarthritis than in healthy donors. Interestingly, the high dominance in the initial sample of one patient of *Prevotella*, a genus previously linked to spondyloarthritis, resulted in the most differential host response upon 16 h host-microbe coincubation. While future research should further focus on inter-individual variability by using gut microbiota from a large cohort of patients, this study underscores the importance of the gut microbiota during the SpA disease course.

## Introduction

Spondyloarthritis (SpA) is a group of chronic inflammatory joint diseases that can affect both axial (the spine and sacroiliac joint) and peripheral joints (upper and lower limbs)^1^. The multifactorial pathogenesis is not entirely understood but involves genetics, immune responses and environmental factors, including gut microbiota^2,3^.

An association between gut and joint inflammation has long been established, however, the mode of action behind the association is not clear. Microscopic and subclinical gut inflammation without overt gastrointestinal symptoms is observed in 50% of SpA patients, is associated with higher disease activity in axial SpA and is a risk factor for developing inflammatory bowel disease (IBD)^4,5^. Up to 10% of SpA patients develop IBD on the long term and vice versa, 20% of IBD patients have SpA^6^. Genome-wide association studies (GWAS) revealed that perturbations in type 3 immunity and the IL-17/IL-23 axis are central in both SpA and IBD pathogenesis (e.g. polymorphisms in *IL-23R* gene)^7–9^. Under healthy, physiological circumstances, type 3 immune cells (such as innate lymphoid cells, CD8 + and CD4 + T cells and innate-like T cells) are involved in maintaining the gut epithelial barrier integrity by producing IL-17 and IL-22, induced by IL-23^8^. IL-23 is primarily produced by macrophages and dendritic cells in response to the recognition of microbes and microbial products through pattern recognition receptors^10^. In SpA patients, however, type 3 innate lymphoid cells were found to be enriched in the gut, peripheral blood and synovial fluids^11^. Moreover, the gut epithelial barrier was found to be compromised, suggesting that translocation of gut microbiota and microbial products to the bloodstream can alter systemic immune responses in SpA^12^. The mode of action of type 3 immune cells and the involved pathways in the joints are unclear. Besides, no consensus is reached about specific associations of immune cells at distinct sites in the gut and joints^2^.

There is accumulating evidence suggesting a link between the gut microbiome and SpA pathogenesis^13^. HLA-B27 transgenic rats, an animal model reflecting human SpA by spontaneously developing both gut and joint inflammation, are symptom-free when raised in germ-free conditions due to the absence of interactions between innate immunity and gut microbes^14^. Subsequent colonization of germfree rats with *Bacteroides vulgatus* is sufficient to induce inflammation, indicating the important interplay between host immune response and microbiota in the SpA pathogenesis^15^. This indicates that, next to IL-23 production in response to microbes, in vitro models mimicking host-microbe interactions in the gut during SpA, require the inclusion of myeloid cells (monocytes, macrophages, dendritic cells and granulocytes)^16^. 16S rRNA gene sequencing revealed differences in gut microbiota composition between HLA-B27 transgenic and wild-type animals. Transgenic animals displayed, even in the absence of gut inflammation, an increase in *Prevotella* spp. and a decrease in *Rikenellaceae*, attributing differences in community composition to HLA-B27-status^17^. It is therefore proposed that microbial dysbiosis, a change in microbial diversity and community composition that is associated with a negative impact on host health^18^, contributes to or aggravates gut and joint inflammation, yet it remains unclear whether dysbiosis is a cause or consequence of inflammation^19,20^. A reduced microbial diversity is commonly observed in SpA patients compared to healthy controls^8,21^. Reduced diversity can be a consequence of the gut pro-inflammatory status that creates an oxidative milieu and limits the survival of strictly anaerobic species^20^. Vice versa, a dysbiosis resulting from the loss of short-chain fatty acid (SCFA) producing species might trigger inflammation as SCFA confer anti-inflammatory health benefits to the host^20,22^. Indeed, recent metagenomic studies revealed a decrease in SCFA producing species, such as *Faecalibacterium prausnitzii*, *Roseburia inulinivorans* and *Coprococcus catus* in SpA patients compared to healthy controls^23–25^. *Ruminococcus gnavus*, on the other hand, is enriched in SpA compared to healthy siblings and unrelated controls^26^. Remarkably, the highest abundance was present in patients with an IBD history, independent of disease status (active vs. remission). *R. gnavus* has been linked with Crohn’s disease before and is known for its mucin degrading capacity and pro-inflammatory role^27^. By exposing the epithelial barrier to other invasive and adherent bacteria, *R. gnavus* may thus trigger inflammation^26,27^.

**Figure 1:**
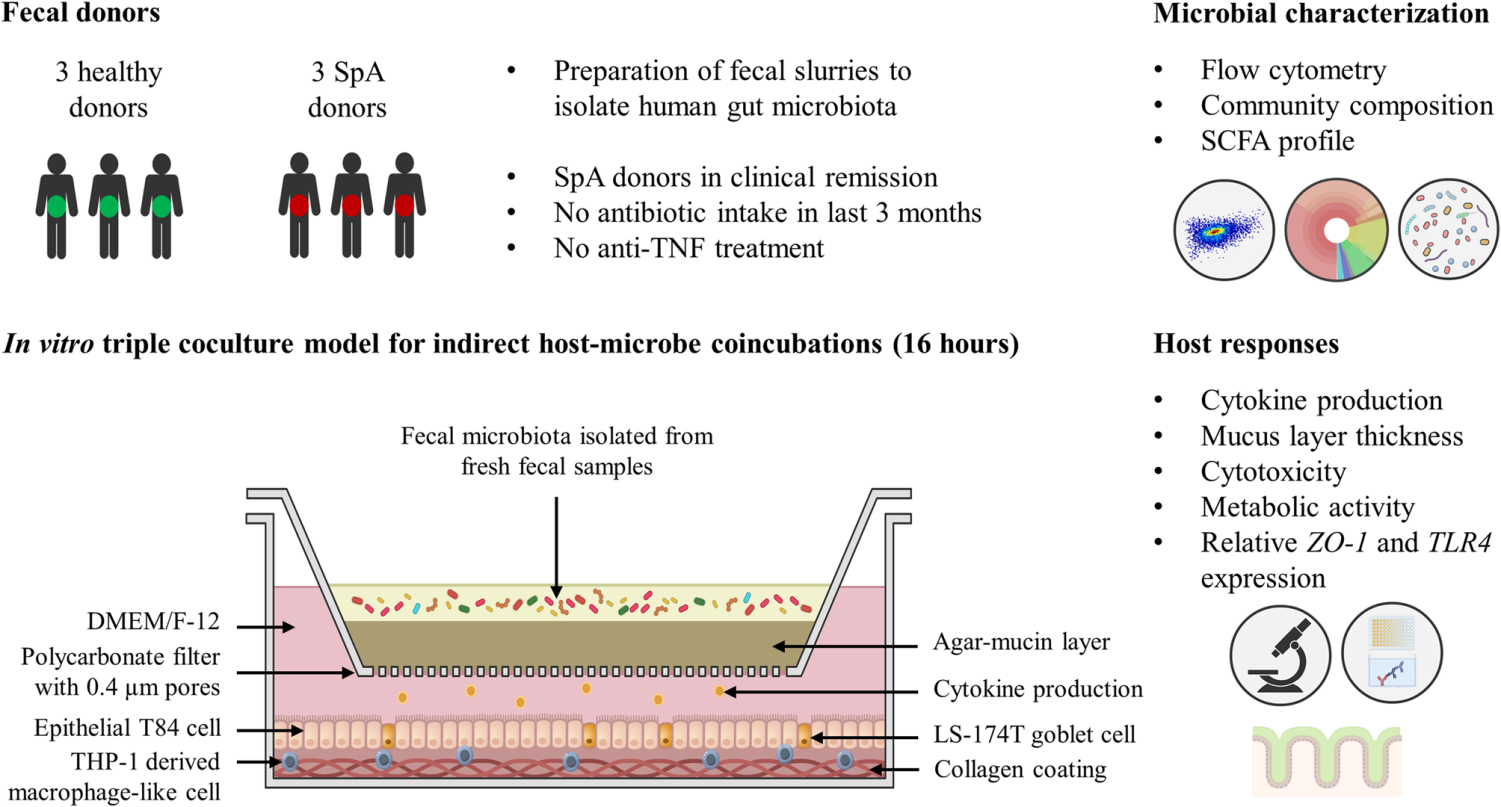
Schematic representation of the experimental approach. In vitro triple coculture figure was adapted from Beterams et al.^28^.

Despite several attempts to explain the inflammation on the gut-joint axis, it is unclear to what extent the gut microbiota is participating in the onset of gut and/or joint inflammation^2^. Moreover, microbiome contributions are difficult to assess in vivo due to the multifactorial disease complexity. We therefore decided to isolate human gut microbiota and evaluate its mechanistic impact on the host in a genetically stable in vitro colon model. We coincubated the fecal microbiota from healthy and SpA donors in a previously characterized in vitro triple coculture model (a coculture of chemically-induced immune-like, goblet and epithelial colon cells)^28^. The triple coculture model was previously^28^ benchmarked and validated against an epithelial T84 monolayer cell model, which revealed that triple cocultures produce thicker mucus layers, morphologically organize in networks and respond to the exposure of human-derived gut microbiota samples via pro-inflammatory cytokine production. The addition of the probiotic *Lactobacillus rhamnosus* GG to assess the immunomodulatory capacity in the triple coculture model demonstrated that pro-inflammatory cytokine responses, based on transcriptomic microarray analyses, were slightly suppressed^28^. In this research, we assessed whether a differential immune response was evoked in the triple coculture model based on the health status of the fecal donor (Fig. 1).

## Results

### Triple cocultures exhibit an elevated donor-dependent pro-inflammatory tone in response to SpA microbiota

**Figure 2:**
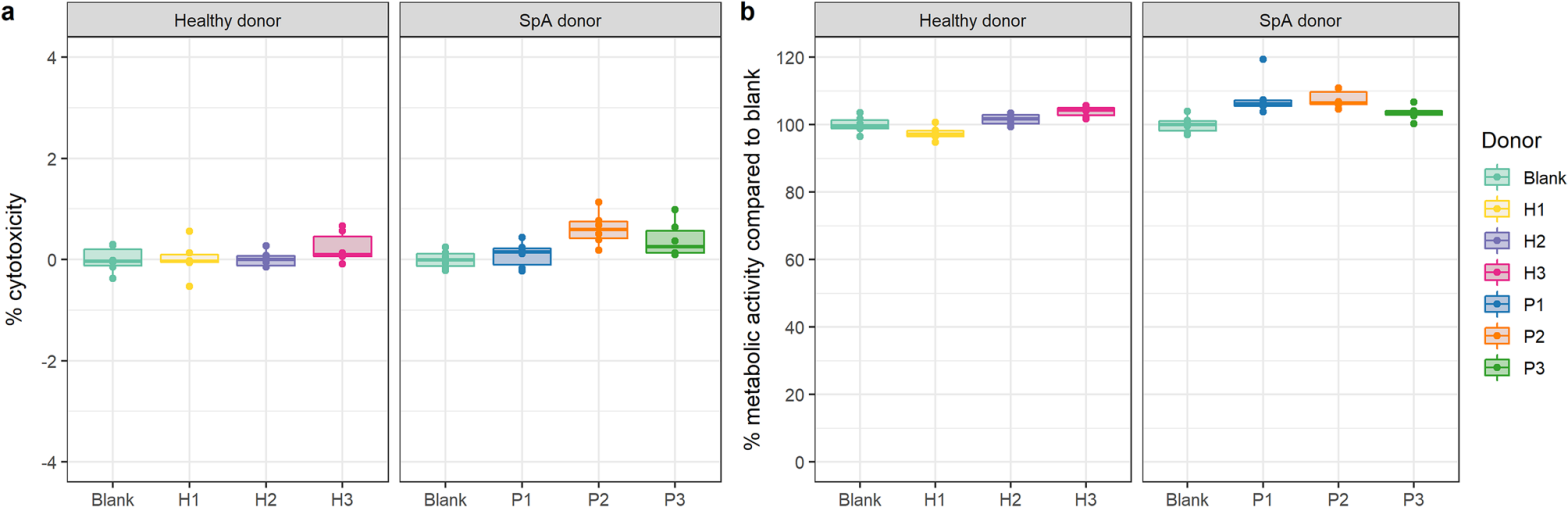
Triple cocultures are robust against exposure to fecal microbiota from healthy and SpA donors. (**a**) Cytotoxic stress as measured by LDH assays. (**b**) Metabolic activity compared to the blank condition (not exposed to bacteria) as measured by a resazurin reduction assay (n = 6).

The fecal microbiota of three healthy and three SpA donors was coincubated for 16 h in an in vitro triple coculture model for the colon. The coincubation time of 16 h was previously established^28^ and aimed for representative viable bacterial cell counts comparable to in vivo colon conditions (10^5^–10^6^ bacterial cells/mL mucus)^29^, while minimizing the possibilities to induce cytotoxicity in host cells. In addition, this timeframe is comparable to the colonic transit time of luminal material (16–24 h)^30^. As microbiota may elicit a stress response in the triple cocultures, we first performed a quality control step to verify whether any of the applied fecal microbiota samples induced cytotoxic stress. We assessed cytotoxicity by measuring lactate dehydrogenase (LDH) release in the basolateral coculture medium and observed that for all conditions the cytotoxicity remained below 1%, thereby confirming the absence of cytotoxic stress (Fig. 2a). Secondly, we assessed the metabolic activity of host cells since changes in activity can result from mild cytotoxicity (e.g. oxidative stress, exposure to bacterial metabolites and intestinal alkaline phosphatase production) which may, in turn, modify the production and secretion of proteins of interest (e.g. cytokines, mucus). Metabolic activities were determined by measuring the reduction of resazurin to the fluorescent resorufin and by expressing values as percentages compared to the blank condition (not exposed to bacteria) (Fig. 2b). With metabolic activities varying between 97.40 ± 2.02 and 108.05 ± 5.68%, none of the applied microbiota samples modified metabolic activities below 90% or above 110% as compared to the blank condition. We therefore concluded that measured cytokine and mucus endpoints in host-microbe coincubations were not confounded by any cytotoxic side effects and could be used to assess host responses to donor-specific fecal microbiota samples.

**Figure 3:**
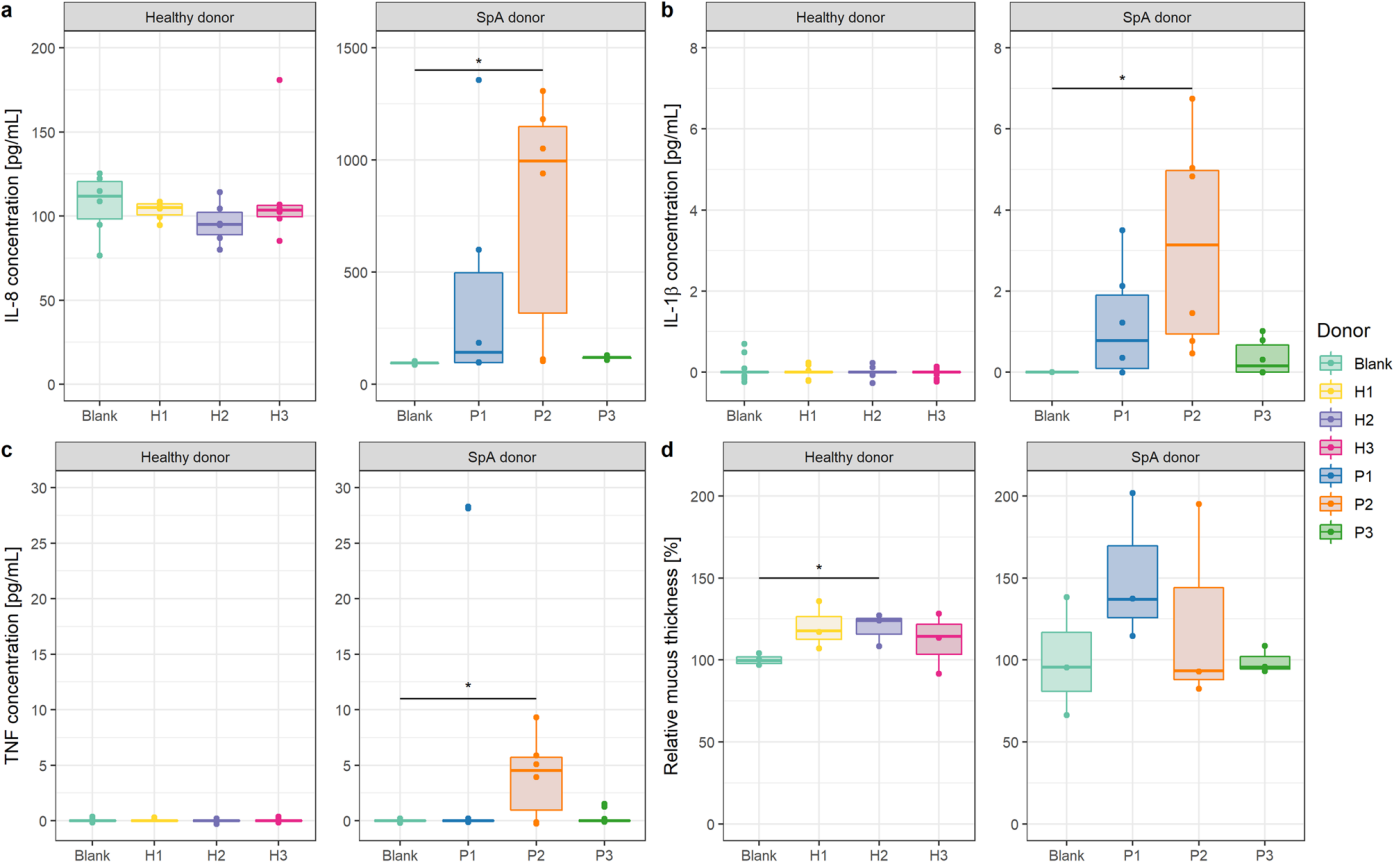
Triple cocultures coincubated with fecal microbiota from SpA patients respond in a more heterogeneous donor-dependent manner compared to healthy donors. Cytokine and mucus productions are benchmarked against the blank condition (not exposed to bacteria). (**a**) IL-8 concentration, y-axis displayed on a different scale for healthy and SpA donors (n = 6). (**b**) IL-1β concentration (n = 6). (**c**) TNF concentration (n = 6). (**d**) Relative MUC2 mucus production compared to the blank control condition, not exposed to bacteria (n = 3). *p < 0.05.

None of the three healthy donors induced significant changes in IL-8 (p = 0.38), IL-1β (p = 0.14) or TNF (p = 0.25) production in triple cocultures as compared to each other or to the blank condition (Fig. 3a–c). For SpA donors, in contrast, more variability was observed between donors and within donors, as displayed by the large technical variability between replicates. Patient sample 1 (P1) tended to increase IL-8 production (p = 0.072) compared to the blank condition, yet no significant difference was found due to high technical variation (Fig. 3a). IL-1β (p = 0.12) and TNF (p = 0.31) production were not affected by fecal microbiota of P1 compared to the blank (Fig. 3b,c). P2 microbiota significantly increased the production of pro-inflammatory cytokines. P2 increased IL-8 production to 782.50 ± 537.51 pg/mL compared to the blank condition (94.75 ± 5.18 pg/mL, p = 0.0036) (Fig. 3a). IL-1β production increased to 3.21 ± 2.65 pg/mL compared to the blank condition (0 ± 0 pg/mL, p = 0.0017) and also the TNF production increased to 4.07 ± 3.67 pg/mL compared to blank (0 ± 0 pg/mL, p = 0.013) upon coincubation with P2 (Fig. 3b,c). P3 tended to increase IL-8 production from 94.75 ± 5.18 pg/mL (blank condition) to 118.29 ± 7.29 pg/mL, yet significance at the 5% level was not reached (p = 0.057) (Fig. 3a). IL-1β (p = 0.22) and TNF (p = 0.48) production, on the other hand, were not modified by P3 microbiota (Fig. 3b,c). When comparing all healthy versus all SpA donors, a significant increase in IL-1β production was found in SpA donors compared healthy donors (p < 0.001) (Fig. 3b).

Since pro-inflammatory cytokines can be present in fecal samples, we also quantified IL-8, IL-1β and TNF concentrations in the fecal slurries used at time point zero (Supplementary Fig. S1). Both IL-1β and TNF were only detected in SpA donors, with concentrations of 43.19 ± 4.46 pg/mL IL-1β and 47.40 ± 2.12 pg/mL TNF in P1, 33.50 ± 3.57 pg/mL IL-1β and 17.62 ± 1.36 pg/mL TNF in P2 and 13.04 ± 0.53 pg/mL IL-1β in P3 (no TNF was detected in P3). With respect to IL-8, low concentrations varying between 4.76 ± 0.07 and 5.70 ± 0.57 pg/mL were found in both healthy and SpA donors. Our experimental approach, however, ensured that only adhered microbiota remained present during the 16 h coincubation with triple coculture host cells while the remaining fecal water with remnant cytokines was washed away. Any modulation of cytokine levels in our experimental setup was thus a result from a response of the cells to the human-derived microbiota, and not from residual cytokines in the fecal sample.

Microbiota can impact the production of mucus in the gut. In our model, we assessed the relative mucus production by triple cocultures under the influence of fecal microbiota compared to the blank condition (Fig. 3d). We observed a trend in increasing mucus production upon coincubation with fecal microbiota of two healthy donors (120.08 ± 14.04% for H1, p = 0.076 and 119.34 ± 10.48% for H2, p = 0.041) compared to the blank condition (100 ± 4.07%), while H3 did not change (112.10 ± 18.44%, p = 0.26). In SpA donors, mucus production displayed more variability between donors and did not change significantly compared to the blank condition (p = 0.44).

### Microbial community composition from SpA background displays more inter-individual variation than that from healthy donors

**Figure 4:**
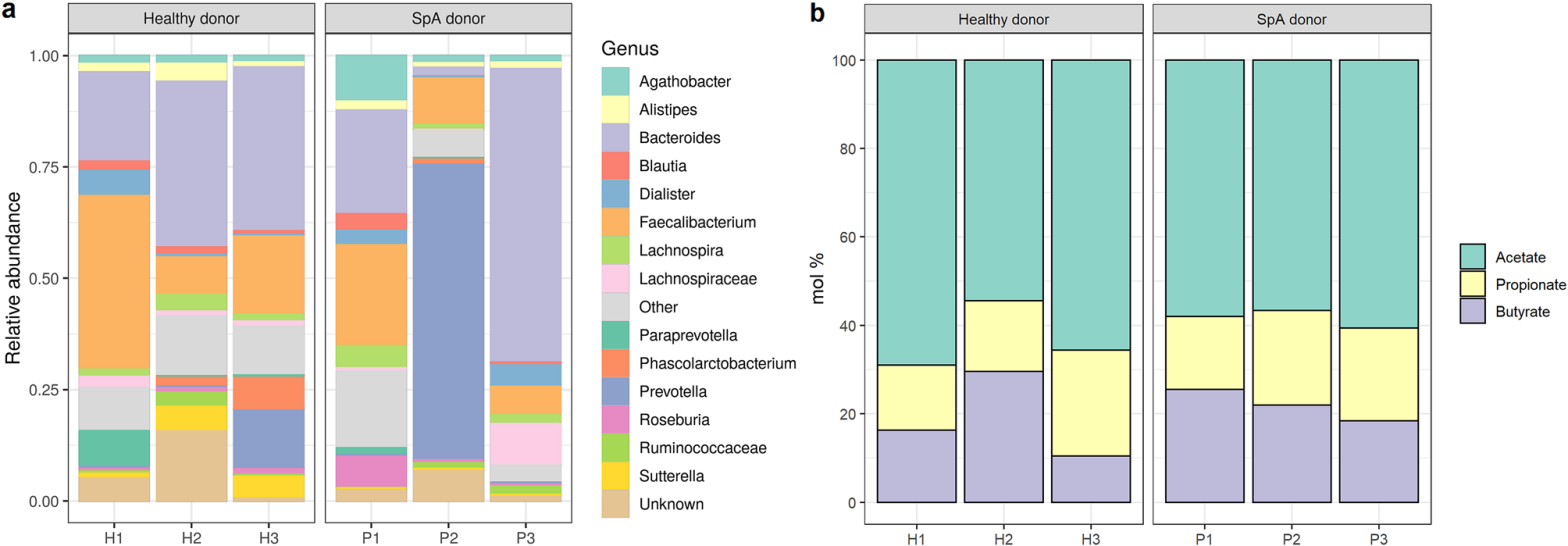
Microbial characterization of fecal slurries at time point zero of host-microbe coincubations. (**a**) Relative abundance of the microbial community composition at genus level. The 15 most abundant genera per donor are displayed. Less abundant genera are grouped into ‘Other’. (**b**) Short-chain fatty acid (SCFA) composition in mol %.

The relative community composition at genus level, showing the 15 most abundant genera, revealed that the microbiota composition was similar in healthy donors (Fig. 4a). In SpA donors, we observed more variability between the different donors. The composition of P1 was similar to the healthy donors with large relative abundances of *Bacteroides* (23.69%) and *Faecalibacterium* (23.02%). P2 and P3 on the other hand, were dominated by Gram-negative genera. P2 was dominated by *Prevotella* (66.68%) and P3 was dominated by *Bacteroides* (66.29%). Recently, *Dialister* has been suggested as a microbial marker for disease activity in SpA^31^. *Dialister* is an obligately anaerobic or microaerophilic, nonmotile, nonspore-forming, non-fermentative, small Gram-negative coccobacil producing variable amounts of acetate, lactate, and propionate as metabolic end products^32^. In our SpA donors, P1 and P2 were in clinical remission, relative abundances of *Dialister* were 2.84% in P1 and 4.81% in P3, while no *Dialister* was found in P2. Also in two healthy donors, we observed *Dialister* (5.56% and 0.22% in H1 and H2 respectively). *Phascolarctobacterium*, another genus known for its propionate-producing capacity, was detected in H2 (2.35%), H3 (7.33%) and P2 (1.16%) and showed higher abundances in donors with a lower abundance of *Dialister* and vice versa. The high *Phascolarctobacterium* levels in H3 corresponded to a high propionate proportion in the fecal slurry of H3 at time point zero (Fig. 4b). Analysis of the alpha diversity (inverse Simpson index) and beta diversity (using a PCoA plot based on Bray–Curtis distances) of the metataxonomic analysis of the 16S rRNA marker gene, did not reveal differences between healthy and SpA donors (Supplementary Fig. S2). Short-chain fatty acid (SCFA) compositions were quantified as these can have an impact on the intestinal barrier function. However, based on the relative propionate (18.22 ± 5.00% and 19.61 ± 2.72%, for healthy and SpA donors, respectively), acetate (62.97 ± 7.58% and 58.39 ± 2.02%, for healthy and SpA donors, respectively) and butyrate (18.81 ± 9.81% and 21.99 ± 3.58%, for healthy and SpA donors, respectively) compositions, it was not possible to discriminate between healthy and SpA donors (p = 0.69, p = 0.37 and p = 0.62 for propionate, acetate and butyrate, respectively) (Fig. 4b). The relative community compositions and intact flow cytometric counts after 16 h coincubation are available in Supplementary Figure S3 and S4. Unfortunately, due to low microbial cell numbers (ca. 4 log intact cells per Transwell insert at 16 h), we could not obtain 16S rRNA gene sequencing data for all donors (no data available for H1, H2 and P3). For H3, a shift in community composition was observed with dominance of genera *Hafnia-Obesumbacterium* and *Escherichia/Shigella* in the first and second replicate, respectively, which was linked with 5.18 ± 1.99 log intact cells per insert. For P1, a shift in dominance to *Escherichia/Shigella* was observed for all replicates (8.11 ± 0.19 log intact cells per insert). The same shift was observed for P2, except for one replicate, which was dominated by *Enterococcus* (8.25 ± 0.33 log intact cells per insert).

### Phenotypic clustering differentiates between healthy and SpA donors

**Figure 5:**
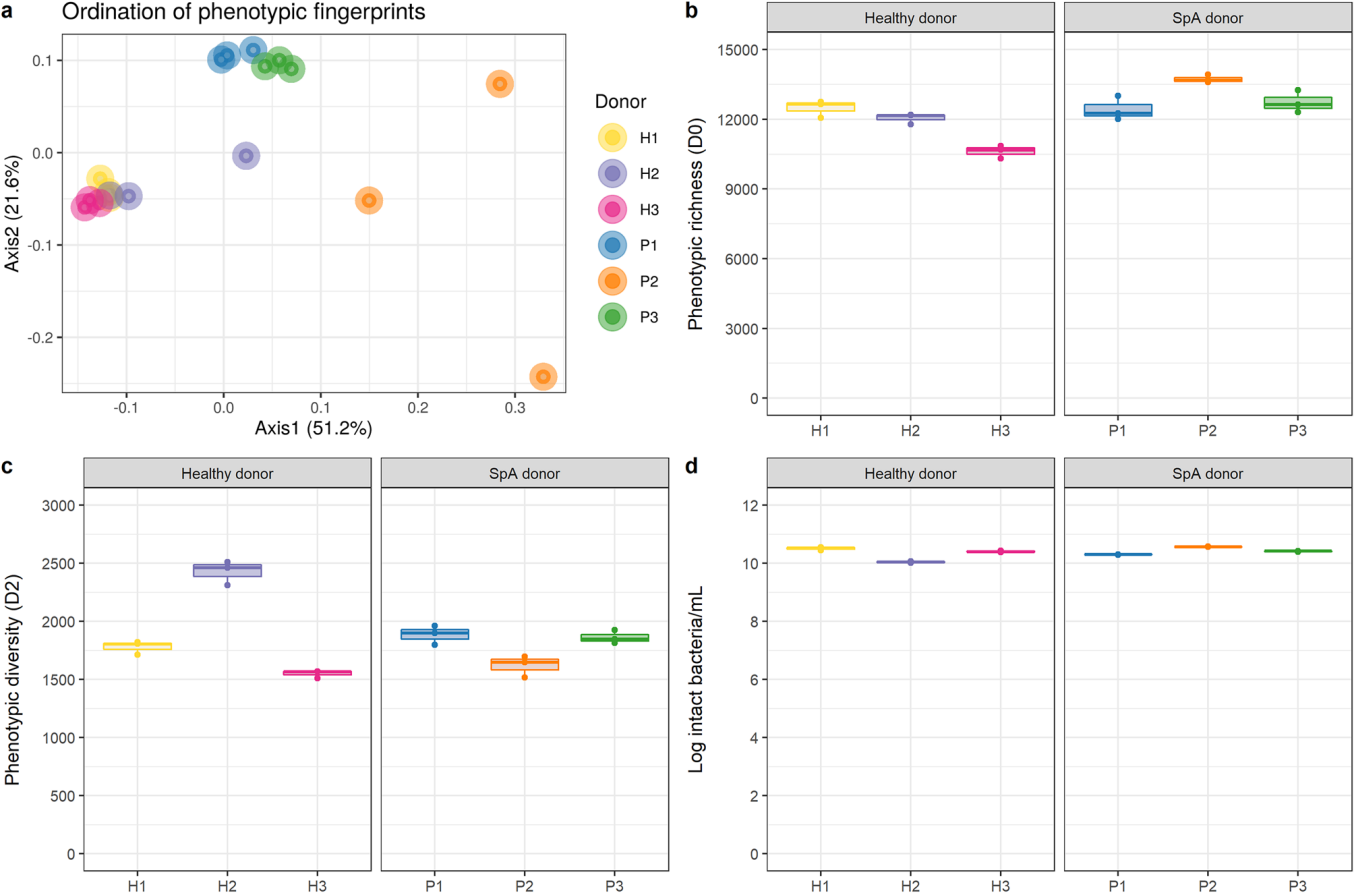
Flow cytometry fingerprinting and cell counting of fecal slurries at time point zero. (**a**) Phenotypic beta diversity displayed in a PCoA plot using Bray–Curtis distances. (**b**) Phenotypic richness (D0). (**c**) Phenotypic alpha diversity (D2). (**d**) Intact bacterial cell counts (n = 3).

Besides a metataxonomic analysis of the 16S rRNA marker gene, timepoint zero microbial communities were also characterized with flow cytometry fingerprinting in order to profile the phenotypic diversity^33^. Phenotypic beta diversity assessment with a PCoA plot based on Bray–Curtis distances revealed that healthy fecal donors clustered together and apart from SpA donors (Fig. 5a). Samples from P2 were distinct from the other SpA donors and also stood out in terms of community composition, which was largely *Prevotella* dominated, and cytokine response, which was increased compared to the blank. Additionally, and in line with the highly variable cytokine measurements, P2 replicates were more scattered across the ordination space (Fig. 5a). In contrast to the beta diversity, the alpha diversity measures (D0-richness or D2- diversity based on inverse Simpson index) did not show a consistent divergent trend between healthy and SpA fecal donors (Fig. 5b,c). Similarly, healthy and SpA individuals could not be distinguished based on fecal intact cell counts which all ranged between 10.40 ± 0.03 and 10.57 ± 0.01 log cells/mL, respectively (Fig. 5d).

### Inter-individual response in ZO-1 and TLR4 expression in triple cocultures exposed to gut microbiota

**Figure 6:**
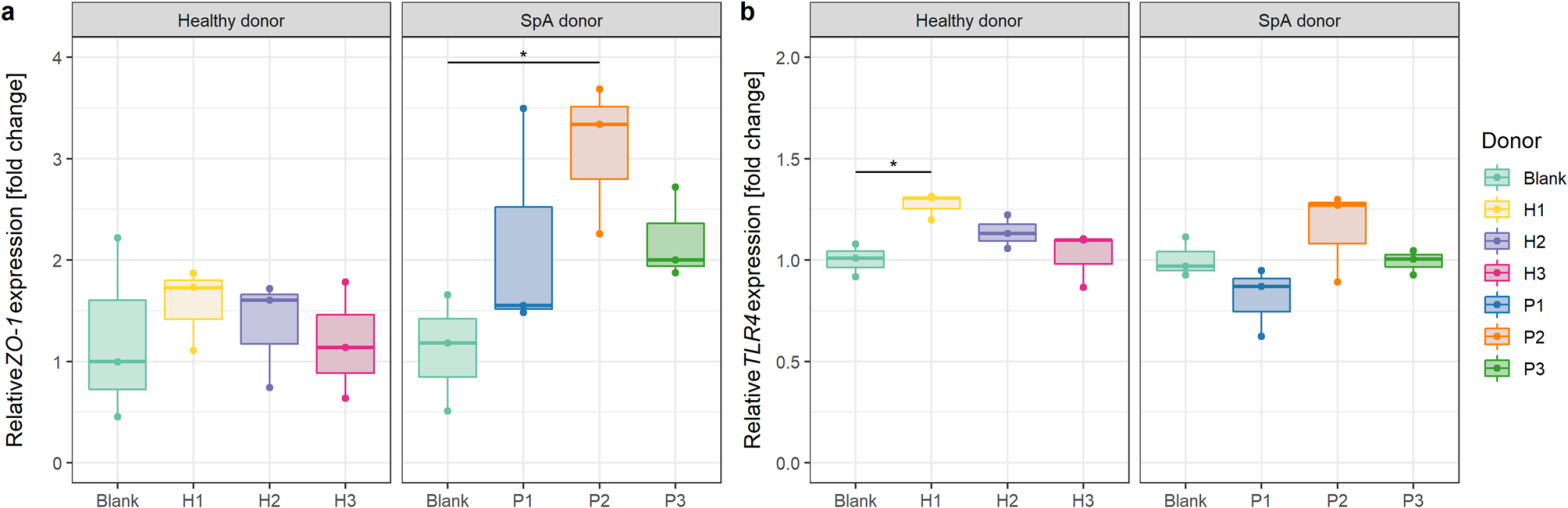
Relative mRNA expression of (**a**) *ZO-1* and (**b**) *TLR4* in triple cocultures exposed to fecal microbiota compared to blank triple cocultures (not exposed to microbiota) (n = 3). *p < 0.05.

The exposure of triple cocultures to fecal microbiota of healthy donors did not change *ZO-1* mRNA expression (p = 0.79) (Fig. 6a). In the case of SpA donors, P2 significantly increased *ZO-1* expression (3.09 ± 0.75 fold change, p = 0.048), while for P1 and P3 no significant differences were found (p = 0.26 and p = 0.19, respectively) (Fig. 6a). The expression of *TLR4* on the other hand, was not impacted by SpA donors (p = 0.15) nor by H2 and H3 (p = 0.39 and p = 0.99, respectively); only healthy donor H1 slightly increased *TLR4* expression (1.27 ± 0.06 fold change, p = 0.049) (Fig. 6b).

## Discussion

We coincubated fecal microbiota samples of healthy and SpA donors in an in vitro triple coculture model for the colon to assess whether host-microbe interactions are affected by the health status of the donor. We were able to study the isolated effect of the gut microbiota on host cells by using genetically stable cell lines, thus eliminating the genetic background of hosts while focussing on the microbiota impact.

Pro-inflammatory cytokine responses of triple cocultures displayed a high technical variation. This can be explained by the combined variability of a dynamically changing host and microbial compartment. Cytokine responses also yielded higher inter-individual variation and an increased trend in SpA compared to healthy donors. Since we can exclude the possibility that measured cytokines were originating from the applied fecal slurries, we can assume this is the result of the differences in microbial community composition in SpA donors. It is important to note, however, that the host cell responses (pro-inflammatory cytokines, mucus production and relative *TLR4* and *ZO-1* expression levels) we observed upon 16 h coincubation cannot be ascribed to the microbial community compositions and flow cytometry results of the fecal slurries measured at time point zero (initial samples). In addition, it is possible that these 16 h host cell endpoints were driven by stochastically-selected facultative anaerobic microbial species due to the aerobic conditions in which the experiments were taking place. For example, *Escherichia/Shigella* and *Enterococcus,* both facultative anaerobic genera, were dominating the microbial community compositions at 16 h and may therefore have impacted the host cell endpoints. Since the conditions of the coculture setup now selectively enrich facultative anaerobes over the coincubation timeframe, in future experiments these conditions can be reconsidered. One possibility is to impose anaerobic conditions on top of the agar-mucin layer by placing a layer that shields the microbial compartment from aerobic conditions, while aerobic conditions in the host compartment can be maintained. Alternatively, one could seek strategies to place the entire setup under anaerobic conditions, while maintaining oxygenated conditions in the host compartment to ensure cell viability. The strategies currently implemented in gut-on-chip models to impose oxygen gradients^34–37^ may be of inspiration for future experimental setups to keep facultative anaerobes at bay. In order to identify which microbial changes are driving host cell endpoints, an assessment of the changes in microbial community composition and functionality is required during the time course of host-microbe coincubations. Inter-individual variation in cytokine responses has previously been linked with specific gut microbiota profiles and functions and the modulation of host responses was mainly attributed to the release of metabolites^38^. Our setup only allowed indirect host-microbe interactions: modulation of cytokine responses is therefore expected to go via secretion of bioactive bacterial molecules/metabolites.

*Prevotella* dominated the initial relative microbial community composition (0 h samples) in P2 and *Bacteroides* in P3. Associations between joint/gut inflammation and *Prevotella* have been reported in other rheumatic diseases as well. In fecal samples of new-onset rheumatoid arthritis patients, the abundance of *Prevotella* was increased and correlated with a decreased abundance in *Bacteroides*^39^. Moreover, the presence of *Prevotella copri* was correlated with rheumatoid arthritis and associated with intestinal dysbiosis and disease susceptibility^39,40^. In contrast, Costello et al. ^41^ identified indicator taxa and found increased abundances in *Lachnospiraceae*, *Ruminococcaceae*, *Rikenellaceae*, *Porphyromonadaceae*, *Bacteroidaceae* and decreased abundances in *Veillonellaceae* and *Prevotellaceae* in the terminal ileum of ankylosing spondylitis patients compared to healthy controls. In HLA-B27 transgenic Lewis rats, *Prevotella* spp. were increased compared to wildtype animals^17^. In transgenic Fischer strains, however, an increased abundance in *Akkermansia muciniphila* was found compared to wildtype animals^40^. Since both of these microbial taxa can degrade mucus, this functionality is suspected to exacerbate inflammation and pro-inflammatory cytokine production by facilitating microbial access to the underlying epithelium^40^.

Besides an increased mucus breakdown, a reduced mucus production may impair the epithelial barrier. In vivo, dysregulation of goblet cell function is one explanation of diminished mucus amounts in a diseased context (e.g. in IBD)^40,42,43^. In our setup, however, goblet cells are not predisposed to genetic disease host backgrounds. In line with this, no significant changes in mucus thickness compared to blank controls were found upon exposure to microbiota isolated from SpA patients. Yet, mucus thickness tended to be consistently higher in triple cocultures exposed to healthy donors (significant in H1 and H2) compared to the blank controls (not exposed to bacteria). This microbiota-induced higher mucus production in healthy individuals is physiologically relevant and represents an important physical separation, limiting contact between microbes and the epithelial barrier in the gut^43,44^. While the P1 microbiota behaved similar to the healthy donors in displaying a tendency to promote mucus production, exposure to microbiota of P2 and P3 failed to stimulate mucus production. This failure to increase mucus thickness may be partially related in P2 to the initial dominant presence (0 h sample) of *Prevotella*, a mucus-degrading pathobiont (e.g. *P. copri*, *P. intestinalis*)^39,45,46^, however, since we did not detect these bacteria at the end of 16 h host-microbe coincubations, we cannot provide evidence that these were involved in degrading mucus. Likewise, P3 had an initial dominant presence (0 h sample) of *Bacteroides*, a genus with species capable of mucin degradation (e.g. *B. thetaiotaomicron*, *B. fragilis*, *B. vulgatus*)^44,47^, however, these bacteria were not detected at the 16 h endpoint, which means that no evidence can be provided to link initially present microbes with mucus degradation upon 16 h host-microbe coincubation. More data concerning the microbial changes during the 16 h coincubation and at the end of the 16 h host-microbe coincubations is required to determine which changes were driving mucus production.

A compromised epithelial barrier integrity related to a decreased expression in tight junction proteins has been found in ileal biopsies of SpA patients compared to healthy ileal biopsies^12^. In our model system, an intact epithelial barrier, not genetically predisposed to SpA, is simulated. This healthy epithelium exhibited an increased expression of *ZO-1* upon indirect contact with microbiota. The increment in expression was larger for SpA than for healthy individuals. Particularly the microbiota of SpA donor P2 resulted in a comparatively large *ZO-1* upregulation, which was significant as opposed to the other donors. We surmise that the increased expression of *ZO-1* and the higher pro-inflammatory cytokine production that we observed in P2, and to a lesser extent in P1 and P3, can be seen as a defensive strategy of the healthy in vitro epithelial barrier in response to the donor-specific SpA microbiota. The fact that limited variation was observed in *TLR4* expression in conditions with higher IL-8 productions, may be related to the timeframe of 16 h coincubation we evaluated, while *TLR4* expression is already induced upon 15 min of LPS stimulation (5 µg/mL) in T84 cells and may reach maximal expression levels at 3 h^48^. In addition, IL-8 production may be induced through other pathways, e.g. the activation of TLR9 that recognizes unmethylated CpG dinucleotides during host-microbe interactions may lead to the activation of NF-κB, a central regulator in many inflammatory pathways, which may, in turn, also induce IL-8 expression^49^.

*Dialister*, a propionate-producing bacteria, was present in both healthy (H1 and H2) and SpA donors (P1 and P3) in the initial fecal slurries (0 h samples) in our study. P3 did not show any signs of intestinal inflammation, but displayed the highest relative *Dialister* abundance from our three SpA donors (4.81%). Previously, *Dialister* has been proposed as a potential microbial biomarker of disease activity in SpA and its abundance was found to be higher in inflamed intestinal biopsies^31^. The discrepancy between our result and the previously published study may be attributed to the different sample types (fecal samples vs. ileal and colonic biopsies) and the limited number of donors we analysed. Interestingly, we noted that lower relative *Dialister* abundances were consistently observed with higher relative abundances of *Phascolarctobacterium*, possibly indicating that these propionate-producing genera are competing for the same functional niche in our samples^44,50^. Based on our data, however, no causal statements of these propionate-producing bacteria in relation to the SpA pathogenesis can be formulated.

The limited number of donors, the high technical variability between replicates, as well as the use of cancer cell lines are the major drawbacks of this study. In addition, this study did not specifically test the in vitro interactions between immune and epithelial cells. Although the expression of specific markers for the colon (*MCT1* and *MS4A12*), for macrophages (*CD68* and *CD11b*) and for the production of mucin type 2 (*MUC2*) were previously shown in the triple coculture model^28^, this may be an interesting perspective to further consider in future studies. We acknowledge that in vitro cell lines can only mimic a share of the innate immune responses occurring in vivo. In this specific study, however, we used the reproducible behaviour of cell lines to our advantage, since it allowed us to assess the inter-individual impact of gut microbiota on host cells, while excluding the host variability.

Overall, we observed more variability in the SpA microbiome and a larger impact on host inflammatory parameters, accompanied with large technical variabilities (especially in P2) compared to healthy donors, while the impact on the gut barrier was limited. In the triple coculture model, the SpA microbiome of P2 elicited an inflammatory response, however, not severe enough to damage the epithelial barrier. This seems to be in accordance with in vivo literature, where chronic inflammation in SpA patients is present, but not (yet) always having an impact on gut barrier dysfunction^11,12^.

To conclude, although our results contain high technical variability, we showed a larger inter-individual impact of gut microbiota isolated from SpA than healthy subjects in a genetically stable and reproducible in vitro model for the colon. Further validation of the in vitro model system is required, however, to monitor the bacterial dynamics during the time course of 16 h host-microbe coincubations. This will allow to assess the system’s utility to link initial microbial compositions to host cell endpoints, which is at this point not (yet) possible. While future research should further focus on (i) the large technical variability by including more replicates and (ii) the inter-individual variability by using gut microbiota from a large cohort of donors, this research underscores the importance of the gut microbiome in the SpA disease course.

## Methods

The fecal microbiota of healthy and SpA donors (Table 1) were separately coincubated in an in vitro triple coculture model for the colon^28^ to assess whether gut microbes have a differential impact on host-microbe interactions depending on the donor health status (Fig. 1). Cytokine secretion, mucus production and cell membrane integrity were followed up as the major host-microbe interaction readouts (Fig. 1). The microbial community was characterized with flow cytometry, 16S rRNA gene amplicon sequencing and SCFA profiling. Cytotoxicity and metabolic activity of host cells were assessed as a quality control.

**Table 1:** Overview of the metadata for healthy and spondyloarthritis (SpA) fecal donors. Bristol stool scale ranges from type 1 (hard and separate lumps) to type 7 (liquid consistency)^52^. Ankylosing Spondylitis Disease Activity Score with C-reactive protein (ASDAS-CRP) to estimate disease activity^59^. Cut-off values: < 1.3 for inactive disease, 1.3–2.0 for low disease activity, 2.1–3.5 for high and > 3.5 for very high disease activity^60^.

Donor type	Donor	ASDAS-CRP	Age	Sex	Bristol stool scale
Healthy	H1	NA	26	Male	Type 4
Healthy	H2	NA	43	Male	Type 4
Healthy	H3	NA	27	Male	Type 5
Axial SpA	P1	1.1	22	Male	Type 6
Axial SpA	P2	1.1	54	Male	Type 5
Axial SpA	P3	2.2	29	Male	Type 5

### Human cell lines

The T84 cell line (CLS 300,354) and LS-174 T cell line (CLS 300,392) were obtained from Cell Lines Service (Eppelheim, Germany). T84 cells were grown in Dulbecco’s modified Eagle medium/Nutrient Mixture F-12 (DMEM/F-12) containing 15 mM HEPES buffer (Gibco, Thermo Fisher Scientific, Merelbeke, Belgium), supplemented with 10% (v:v) heat-inactivated fetal bovine serum (FBSi) (Greiner Bio-One, Kremsmünster, Austria) and 1% (v:v) antibiotic antimycotic solution containing penicillin, streptomycin and amphotericin B (Merck, Overijse, Belgium). LS-174 T cells were grown in minimal essential medium (MEM) containing Earle’s balanced salts (Gibco, Thermo Fisher Scientific, Merelbeke, Belgium), supplemented with 1% (v:v) non-essential amino acids solution (Gibco, Thermo Fisher Scientific, Merelbeke, Belgium), 10% (v:v) FBSi and 1% (v:v) antibiotic antimycotic solution. The THP-1 cell line was obtained from the European Collection of Authenticated Cell Cultures (ECACC 88,081,201, Public Health England, London, United Kingdom). THP-1 cells were grown in Roswell Park Memorial Institute (RPMI) 1640 with 2 mM GlutaMAX™ (Gibco, Thermo Fisher Scientific, Merelbeke, Belgium) supplemented with 10% (v:v) FBSi and 1% (v:v) antibiotic antimycotic solution. THP-1 cells were routinely grown in suspension in a lateral 3D rotating wall vessel (RWV) bioreactor (Synthecon, Houston, USA). Passages between 48–67, 46–65 and 20–39 of respectively T84, LS-174 T and THP-1 cells were used and cells were confirmed mycoplasma-free prior to setting up experiments using the MycoAlert Mycoplasma Detection kit (Lonza, Basel, Switzerland). All three cell types were grown at 37 °C, 10% CO_2_ and 90% relative humidity.

### Fecal samples

Fecal donations of three healthy donors (H1, H2 and H3) and three axial SpA patients (P1, P2 and P3) were collected in air-tight containers containing an AnaeroGen sachet (Oxoid, Hampshire, UK) to create anaerobic environments and stored at 4 °C until further processing (< 24 h). Fecal slurries of 20% (w:v) were prepared as described by De Boever et al. ^51^ in autoclaved anaerobic phosphate buffer (0.1 M, pH 6.8) supplemented with 1 g/L sodium thioglycolate (Sigma-Aldrich, Overijse, Belgium) as reducing agent. None of the donors received anti-tumor necrosis factor (TNF) treatment or antibiotics three months prior to donation. Donor characteristics (age, sex and Bristol stool scale^52^) are listed in Table 1. Short-chain fatty acids (SCFA) of the fecal slurries were extracted using diethyl ether and measured with gas chromatography as described by Andersen et al. ^53^. Permission for analysis and in vitro coincubations of human fecal material with human cell lines was obtained from the ethical committee of Ghent University Hospital under project number BC-05539 (EC/2019/1239). The research was performed in accordance with the relevant guidelines and regulations. Informed consent was obtained from all participants.

### Seeding of triple cocultures in collagen type I coated plates

A collagen type I coating was prepared in the basolateral side of 24-well plates (Corning Incorporated, New York, USA) by adding 400 µL/well from a 1% collagen type I solution from rat tail (4.5 mg/mL, Sigma-Aldrich, Overijse, Belgium) in cell culture grade H_2_O (Gibco, Thermo Fisher Scientific, Merelbeke, Belgium) and incubated overnight at 4 °C. Excess fluid was removed and the surface was left to dry overnight. The collagen coating was washed with 1 mL/well Dulbecco’s phosphate-buffered saline (PBS, Gibco, Thermo Fisher Scientific, Merelbeke, Belgium) prior to seeding cells. Next, triple cocultures were created by sequentially seeding THP-1, LS-174 T and T84 cells in collagen coated 24-well plates. First, THP-1 cells were seeded in a density of 15 500 cells/cm^2^ in supplemented RPMI. To differentiate THP-1 monocytes to chemically differentiated M0 THP-1 macrophage-like cells, 100 nM phorbol 12-myristate 13-acetate (PMA, Cayman Chemical, Ann Arbor, USA) was added to the medium for 72 h, followed by 24 h of rest in medium deprived from PMA. Next, LS-174 T and T84 cells were seeded in a 20:80 ratio at a density of 155 500 cells/cm^2^ in supplemented DMEM/F-12. Triple cocultures were grown for 3 days until confluency, subsequently, the cells were refreshed with DMEM/F-12 with FBSi and deprived of antibiotic antimycotic solution and incubated for an additional 24 h prior to the setup of host-microbe coincubations. Host-microbe interactions were assessed by coincubation of triple coculture cells with fecal microbes.

### Coincubations of triple coculture model with fecal microbes to assess host-microbe interactions

The apical part of the host-microbe coincubation was prepared by adding 75 µL of a 0.8% agar (Carl Roth, Karlsruhe, Germany)—5% porcine mucin type II-solution (Sigma-Aldrich, Overijse, Belgium) in dH_2_O onto Transwell polycarbonate filters with 0.4 µm pores (Corning Incorporated, New York, USA). Prior to coincubation, flow cytometry was used to quantify intact and damaged bacterial cell counts in the fecal inocula as described by Van Nevel et al. ^54^. Fecal samples were diluted to 5*10^5^ intact cells/mL in filter-sterilized anaerobic phosphate buffer. Per well, 20 µL bacterial solution was incubated on the solidified agar-mucin layers for 2 h at 37 °C, 10% CO_2_ and 90% relative humidity while 1 mL DMEM/F-12 was added to a basolateral compartment without host cells. As a blank (abiotic control), 20 µL anaerobic phosphate buffer was incubated in the apical compartment. After incubation, planktonic microbial cells were washed away with 100 µL sterile anaerobic phosphate buffer and 20 µL sterile anaerobic phosphate buffer was added on top of the mucin-adhered microbes. Next, basolateral compartments with triple cocultures were refreshed with 1 mL DMEM/F-12 deprived from FBSi and antibiotic antimycotic solution and Transwell filters with bacteria were moved to wells with these refreshed triple coculture host cells. The indirect host-microbe coincubation was maintained for 16 h at 37 °C, 10% CO_2_ and 90% relative humidity. Host-microbe coincubations were performed in six technical replicates (6 wells) per donor and three biological replicates (3 healthy and 3 SpA individuals).

### Flow cytometry

After 16 h of host-microbe coincubations, bacterial samples were detached from agar-mucin layers as described by Tsilia et al. ^55^. In brief, the filters were washed with 100 µL anaerobic phosphate buffer. Next, 100 µL 0.5% Triton X-100 (Sigma-Aldrich, Overijse, Belgium) in anaerobic phosphate buffer was added and incubated for 20 min at 37 °C on a rotary shaker (110 rpm) (Ika, Wilmington, NC, USA). Then, filters were washed three times with 100 µL anaerobic phosphate buffer. The washing fluid was centrifuged for 3 min at 1500 × *g* (Eppendorf 5424, Hamburg, Germany) and the supernatant was discarded. Pellets were dissolved and subsequently diluted in filtered (0.22 µm sterile syringe filter, Sigma-Aldrich, Overijse, Belgium) anaerobic phosphate buffer and measured with an Accuri C6 + flow cytometer (BD, Erembodegem, Belgium), equipped with a blue (488 nm) laser and bandpass filters on the FL-1 (533/30 nm) and FL-3 (670 nm) detectors as described by Van Nevel et al. ^54^. The flow cytometer used Milli-Q water as sheath fluid and the BD Accuri C6 Plus software was used for cell count registration and analysis (BD, Erembodegem, Belgium). Intact and damaged bacterial cell counts were measured in technical triplicate after 20 min incubation of the samples with a 1% SYBR Green I (1000 × diluted, Invitrogen, Carlsbad, USA)/propidium iodide (4 µM final concentration, Sigma-Aldrich, Merelbeke, Belgium) (SGPI) staining solution in DMSO at 37 °C in the dark. As a background control, 0.22 µm filtered phosphate buffer and blank samples from agar-mucin filters were measured. Based on flow cytometry data, phenotypic fingerprints were calculated with the PhenoFlow package (version 1.1.2) in R (version 4.1.1)^33^. Alpha and beta diversity were calculated using the *Diversity_rf()* and *beta_div_fcm()* function, respectively, from the PhenoFlow package. Beta diversity was calculated using Bray–Curtis distances and principal coordinates analysis (PCoA) was used to make ordination plots.

### Microbial community composition

#### DNA extraction

Total DNA was extracted from the pellet of 1 mL fecal slurry by means of bead beating with a PowerLyzer (Qiagen, Venlo, the Netherlands) and phenol/chloroform extraction^56,57^.

#### 16S rRNA gene amplicon sequencing and microbial community composition analysis

10 μL genomic DNA extract was send out to LGC genomics GmbH (Berlin, Germany) for library preparation and V3-V4 region amplicon sequencing on an Illumina Miseq platform with v3 chemistry with the primers 341F (5′-CCTACGGGNGGCWGCAG-3′) and 785Rmod (5′-GACTACHVGGGTATCTAAKCC-3′)^58^. Illumina data was processed using the DADA2 pipeline (dada2 package, version 1.20.0 in R, version 4.1.1) as outlined by https://benjjneb.github.io/dada2/tutorial_1_2.html. In brief, read quality was inspected using the plotQualityProfile function, primers were removed and read lengths were trimmed using the filterAndTrim function with the following arguments: trimLeft = c(0,0), truncLen = c(260,260), maxN = 0, maxEE = c(2,2), truncQ = 2, rm.phix = TRUE. Next, a dereplication step was performed, error rates were estimated and an error model was built. Forward and reverse complements of the reserve reads were then merged (using the mergePairs function), on the condition that an overlap of at least 12 bases exists and that the sequences are identical in the overlap region. An amplicon Sequence Variant (ASV) table was created, chimeras were removed and the reads counts were checked through the pipeline. Taxonomy was assigned using the Silva database v132, the relative abundances of the top 15 genera were selected using the microbiome package and displayed in bar plots using the phyloseq package (version 1.36.0) in R. Alpha diversity of the 16S rRNA marker gene was calculated using the inverse Simpson index, beta diversity was calculated using Bray–Curtis distances and PCoA was used to make ordination plots.

### Metabolic activity

Metabolic and mitochondrial activity of host cells was assessed with a resazurin assay (7-Hydroxy-3H-phenoxazin-3-one-10-oxide sodium salt, Sigma-Aldrich, Overijse, Belgium) after host-microbe coincubations as previously described ^28^. Fluorescence was measured in technical triplicates in black 96-well plates at an excitation wavelength of 540 nm and an emission wavelength of 590 nm (SpectraMax M2 plate reader, Molecular Devices, Brussels, Belgium). As a negative abiotic control, the fluorescence of resazurin in DMEM/F-12, incubated in the absence of host cells was subtracted as a background from all fluorescent values. Metabolic activities were then calculated as percentages compared to blank triple cocultures (not exposed to bacteria).

### Cytotoxicity

The cytotoxicity in triple cocultures was assessed by measuring lactate dehydrogenase (LDH) in the basolateral medium after 16 h of coincubation. Quantification was done with the CyQUANT LDH Cytotoxicity assay kit (Invitrogen, Thermo Fisher Scientific, Merelbeke, Belgium) in technical duplicates according to the manufacturers’ instructions. Absorbances at 490 and 680 nm (background correction) were measured with a Tecan Infinite M Plex plate reader (Tecan, Mechelen, Belgium).

### Cytokine quantification

Fecal slurries at time point zero and basolateral cell culture medium after 16 h of host-microbe coincubation were analysed with enzyme-linked immunosorbent assays (ELISA). Fecal slurries were centrifuged at maximum speed for 5 min and the supernatant was used to determine cytokine concentrations (Supplementary Fig. S1). Basolateral cell culture medium was tested as such. IL-8, IL-1β and TNF were quantified both in fecal slurries and cell culture medium in technical duplicates with human uncoated ELISA kits (sensitivity of 2 pg/mL for IL-8, 0.3 pg/mL for IL-1β and 1 pg/mL for TNF) according to manufacturer’s instructions (Invitrogen, Carlsbad, USA). Absorbances at 450 and 570 nm (background correction) were measured with a Tecan Infinite M Plex plate reader (Tecan, Mechelen, Belgium).

### Determination of MUC2 mucus layer thickness with immunofluorescence

To assess mucus type 2 layer thickness, three wells of host cells per tested donor (and per blank) were fixated (after host-microbe coincubations) in Carnoy’s reagent, consisting of 60% ethanol (≥ 99.9%, Sigma-Aldrich, Merelbeke, Belgium), 30% chloroform (≥ 99%, Carl Roth, Karlsruhe, Germany) and 10% glacial acetic acid (≥ 99.7%, Sigma-Aldrich, Merelbeke, Belgium) for 30 min at 4 °C. After three washing steps, 3% (w:v) BSA in PBS was used as blocking solution overnight at room temperature. Blocking solution was removed, 1 µg/mL MUC2 primary polyclonal antibody (PA5-79,702, Invitrogen, Carlsbad, USA) was added and incubated overnight at 4 °C. After three washing steps, 5 µg/mL Alexa Fluor 488 conjugated secondary antibody, (A-11034, Invitrogen, Carlsbad, USA) was incubated for one h at room temperature. Following three washing steps, imaging was performed with a Nikon A1R confocal microscope equipped with a Plan Fluor 40x/0.6 objective (Nikon Instruments Amsterdam, the Netherlands). In each well, z-stacks (slices every 1.25 µm) of mucus layers were recorded at four different positions. To determine the mucus layer thickness, FIJI software (https://imagej.net/Fiji) was used with an in-house developed script (macro) as described by Beterams et al. ^28^.

### RNA extraction

Triple coculture cells were lysed with LBP lysis buffer (RNA extraction kit, NucleoSpin RNA Plus, Macherey–Nagel, Düren, Germany) and homogenized by transferring the lysates to QIAShredder membranes (Qiagen, Hilden, Germany), followed by centrifugation for 2 min at 20 913 × *g* (Eppendorf 5804 R, Hamburg, Germany). Lysates were stored at -80 °C. RNA was further extracted with the NucleoSpin RNA Plus extraction kit (Macherey–Nagel, Düren, Germany). RNA concentrations were determined with a Denovix DS-11 spectrophotometer (Denovix, Wilmington, USA). Next, 1 µg RNA was treated with DNase I (1 U/µL, Thermo Fisher Scientific, Merelbeke, Belgium) in a total reaction volume of 25 µL, diluted with H_2_O (UltraPure DEPC-Treated Water, Thermo Fisher Scientific) for 30 min at 37 °C. The enzymatic reaction was stopped with 1 µL EDTA (50 mM) and an incubation of 10 min at 65 °C. A260/A280 ratios of all samples were between 1.8 and 2.1 (Denovix DS-11 spectrophotometer).

### Quantitative real-time polymerase chain reaction

Total RNA (30 ng) was converted to single-stranded cDNA using the Reverse Transcriptase Core kit (Eurogentec, Seraing, Belgium). Quantitative real-time polymerase chain reactions (qRT-PCR) were performed in 20 µL, consisting of iTaq universal SYBR Green supermix (Bio-Rad Laboratories, Hercules, USA), 400 nM of each primer (Eurogentec) and 70 ng cDNA using a StepOnePlus real-time PCR system. Primer sequences and efficiencies (percentages, calculated using the formula 10^–1^^/slope^−1) of reference genes, *GAPDH* and *ACTB*, and genes of interest, *ZO-1* and *TLR4*, are available in Table 2. Amplification conditions were: initial denaturation for 2 min at 95 °C, followed by 40 cycles of 15 s denaturation at 95 °C and 1 min combined anneal/extension at 60 °C. Reactions were performed in technical triplicates. The expression of *ZO-1* and *TLR4* were normalized to reference genes *GAPDH* and *ACTB* and expressed as fold changes compared to blank triple cocultures (not exposed to bacteria).

**Table 2:** Primer sequences used for quantitative real-time polymerase chain reactions (qRT-PCR) and their corresponding primer efficiency.

Gene	Forward primer sequence, 5′–3′	Reverse primer sequence, 3′–5′	Efficiency [%]	Reference
*GAPDH*	GGAGTCCACTGGCGTCTTCAC	GAGGCATTGCTGATGATCTTGAGG	92.33	^61^
*ACTB*	CTGGAACGGTGAAGGTGACA	AAGGGACTTCCTGTAACAATGCA	91.97	^61^
*ZO-1*	CGGTCCTCTGAGCCTGTAAG	GGATCTACATGCGACGACAA	98.87	^62^
*TLR4*	AATCTAGAGCACTTGGACCTTTCC	AGAAATCTGGACAGGGACTTGGG	103.29	^63^

### Statistical analysis

Statistical analysis was performed in RStudio (version 3.6.1). Normality of the data was assessed with Shapiro–Wilk tests and QQ-plots. Homoscedasticity of the data was assessed with Levene’s test. When normality and homoscedasticity assumptions were met, multiple groups were compared with one-way ANOVA, followed by Tukey’s post-hoc testing. If assumptions were not met, Kruskal–Wallis was performed with Dunn’s post-hoc testing using Holm’s corrections. Significance was considered at the α = 0.05 level.

## Supplementary Information

Supplementary Information.

## Data Availability

The sequence data has been submitted to the NCBI (National Center for Biotechnology Information) database under BioProject Accession Number PRJNA778184.
