# Supplementary material for: In vitro triple coculture with gut microbiota from spondyloarthritis patients is characterized by inter-individual differences in inflammatory responses
Source: Sci Rep. 2022 Jun 21;12:10475. doi: 10.1038/s41598-022-13582-7 (PMC9213446; doi:10.1038/s41598-022-13582-7)
Supplement: Supplementary file 1 — Supplementary Information. [file 41598_2022_13582_MOESM1_ESM.pdf]

# Supplementary information

# *In vitro* triple coculture with gut microbiota from spondyloarthritis patients is characterized by inter-individual differences in inflammatory responses

Annelore Beterams[1], Marta Calatayud Arroyo[1,†], Kim De Paepe[1], Ann-Sophie De Craemer[2,3], Dirk Elewaut[2,3], Koen Venken[2,3], Tom Van de Wiele[1,†,*]

[1]Center for Microbial Ecology and Technology (CMET), Department of Biotechnology, Ghent University, Ghent, Belgium

[2]Department of Internal Medicine and Pediatrics, Division of Rheumatology, Ghent University Hospital, Ghent, Belgium

[3]Unit for Molecular Immunology and Inflammation Unit, VIB-UGent Center for Inflammation Research, Ghent, Belgium

[†]These authors equally contributed to this work.

*Corresponding author:

Tom Van de Wiele, Center for Microbial Ecology and Technology (CMET), Coupure Links 653 Building A, 9000 Ghent, Belgium. Email: Tom.VandeWiele@UGent.be

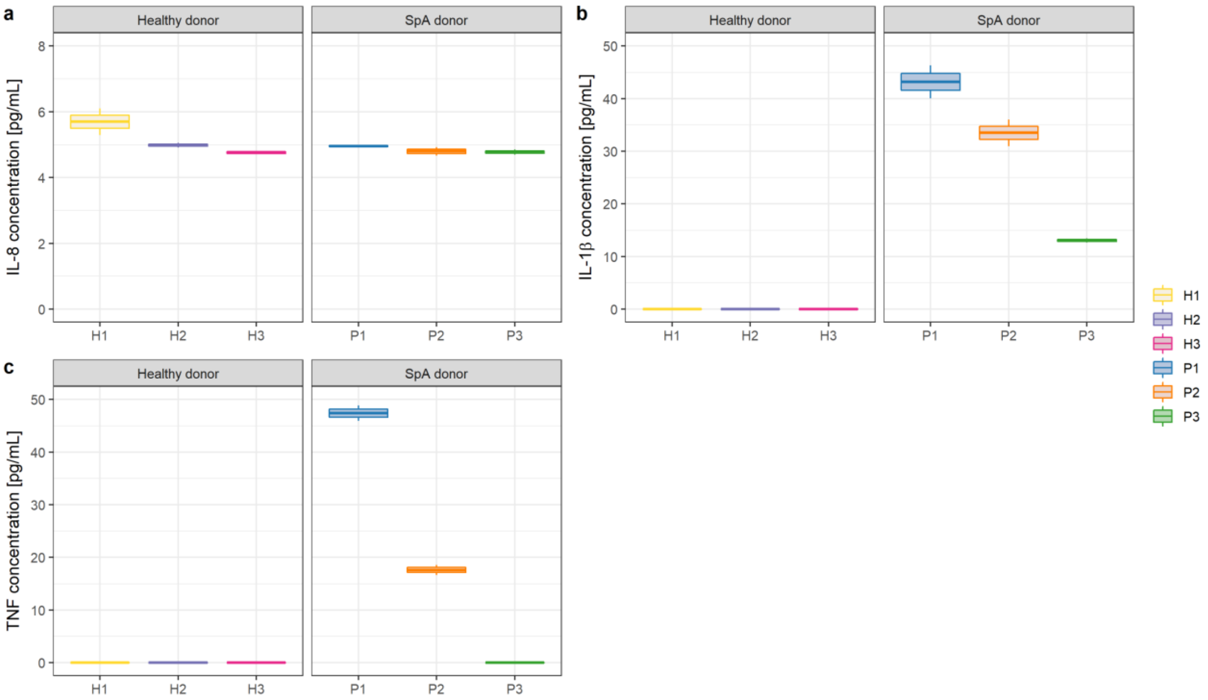


**Supplementary Figure S1** Pro-inflammatory cytokine concentrations in fecal slurries at time point zero of host-microbe coincubations. (**a**) IL-8 concentration. (**b**) IL-1β concentration. (**c**) TNF concentration (n=2).

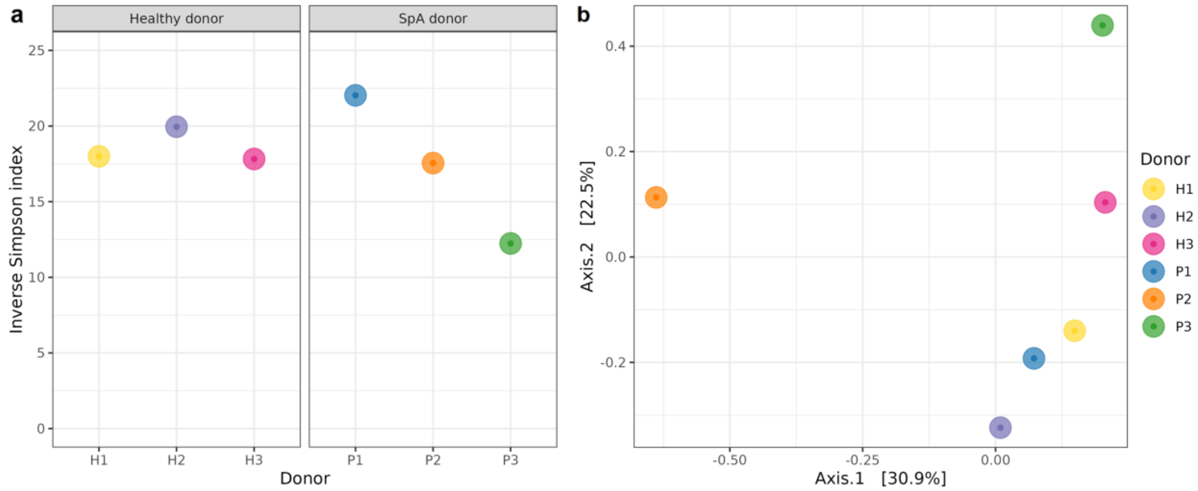


**Supplementary Figure S2** Diversity measures in 16S rRNA gene sequencing samples of fecal slurries at time point zero of host-microbe coincubations. (**a**) Alpha diversity based on the inverse Simpson index. (**b**) Beta diversity displayed in a PCoA plot using Bray-Curtis distances (n=1).

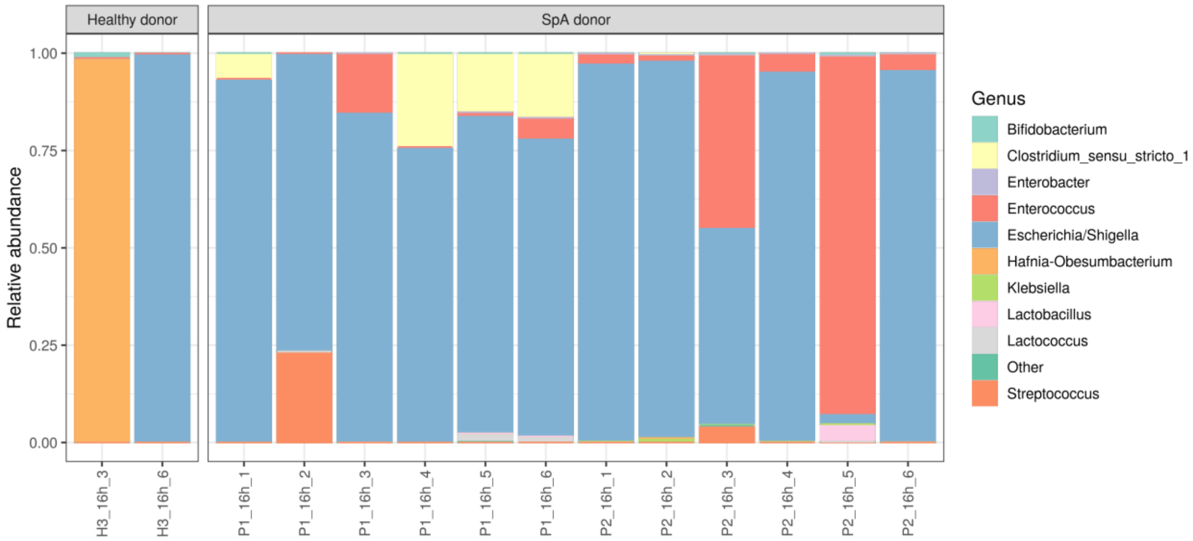


**Supplementary Figure S3** Relative abundance of the community composition at genus level in apical samples after 16 h host-microbe coincubation, the 10 most abundant genera per donor are displayed. For H3, 2 replicates are available, for P1 and P2, 6 replicates are available. No data is available for H1, H2 and P3 after 16 hours due to the low amount of DNA in these samples.

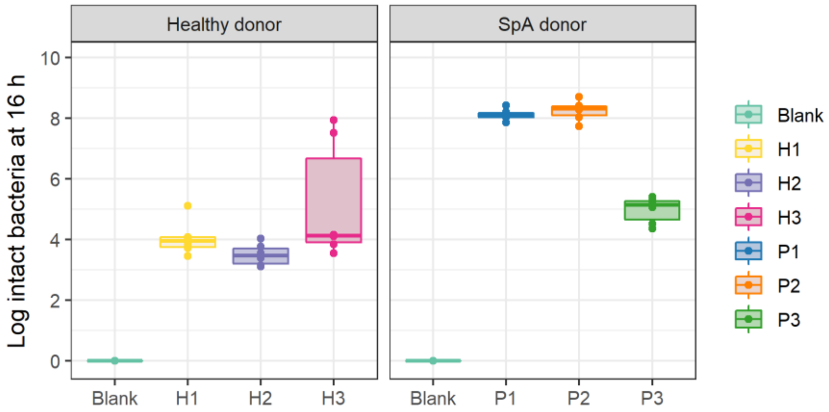


**Supplementary Figure S4** Intact flow cytometric cell counts after 16 h host-microbe coincubation (n=6).
